# Understanding the delays in diagnosis of breast cancer in India using blogs and YouTube videos

**DOI:** 10.1186/s12889-025-24132-x

**Published:** 2025-08-15

**Authors:** Nishita Sarah Thomas, Emma Norris

**Affiliations:** Department of Health Sciences, Brunel University of London, Uxbridge, UK

**Keywords:** Breast cancer, Delayed diagnosis, India, Women, Socio-cultural barriers, Early detection, Public health education, Psychological factors

## Abstract

**Background:**

Globally, breast cancer is the most common cancer among women and is the second leading cause of cancer-related deaths. Breast cancer-related mortality has surged among Indian women, as a significant number of women present at an advanced stage of breast cancer. Early detection has been linked with a higher chance of survival and improved quality of life. This research aimed to assess breast cancer survivor stories of Indian women to identify: (i) why Indian women were diagnosed at an advanced stage of breast cancer, (ii) the barriers that contributed to their delayed diagnosis, and (iii) the level of awareness and perceptions towards breast cancer.

**Methods:**

A qualitative approach using digital web-based methods was used. Existing blogs and YouTube videos were used as secondary data sources. “Breast cancer survivor blogs by Indian women” were searched on Google to identify blogs; and “Breast cancer survivor stories by Indian women” and “Interviews of breast cancer survivors among Indian women” were searched on YouTube to identify videos. 5 blogs and 12 YouTube videos including breast cancer survivor stories of Indian women were analysed using inductive thematic analysis.

**Results:**

A total of 17 Indian breast cancer survivors aged 25–50 were included in this study. Four main themes and seven sub-themes emerged: Delay in recognising or responding to symptoms (sub-themes: lack of knowledge, misinterpretation of symptoms and procrastination), Initial medical misjudgement (sub-themes: dismissal of symptoms by doctors, false reassurance from medical tests), socio-cultural factors (sub-themes: family responsibilities and career responsibilities), and emotional and psychological factors.

**Conclusion:**

This study highlights the complex barriers that contributed to delayed breast cancer diagnosis among Indian breast cancer survivors, through their own personal accounts. These findings highlight the need for enhanced public health education about breast cancer, improved healthcare professional training, and culturally sensitive support to improve early detection, which will ultimately reduce breast cancer mortality and improve the quality of life for Indian women.

**Supplementary Information:**

The online version contains supplementary material available at 10.1186/s12889-025-24132-x.

## Background

Breast Cancer accounts for over 1 in 10 newly diagnosed cancer cases globally and is the second most common cause of cancer-related mortality [1]. In 2020, 2.3 million cases and 658,000 fatalities were due to breast cancer, establishing it as the most prevalent form of cancer worldwide [2]. Epidemiological studies showed that the global disease burden of breast cancer is projected to surge over 2 million cases by the year 2030 [1].

### Breast cancer in India

The incidence of breast cancer has surged in low- and middle-income countries due to changes in lifestyle, reproductive factors, and increased life expectancy [3]. In Indian women, breast cancer has now surpassed cervical cancer as the most common cause of cancer death [4]. Over 145,000 new breast cancer cases are reported annually in India with over 70,000 deaths [5]. 70% of breast cancer in India presents at Stage 3 or 4 [6]: leading to this high death rate [7]. For example, an assessment of female breast cancer diagnosis in one of the largest tertiary-care cancer centres in India between 2014 and 2019 showed that out of 977 patients, only 40 patients were detected with stage I (4%), 326 patients with stage II (33%), 419 with stage III (42%) and 212 with stage IV (21%) breast cancer [8]. Patients with stage I disease had a better survival rate of 93.3%, while those with stage IV disease had a survival rate of 24.5%, with an overall survival rate of 73.8% [9]. Data from India’s National Cancer Registry Programme identified that the areas of Tamil Nadu, Telangana, Karnataka and Delhi had a higher burden of breast cancer than states in the eastern and north-eastern regions [10]. The World Cancer Report 2020 states that early detection and rapid treatment are the most effective interventions for controlling breast cancer [2], with early diagnosis leading to greater survival rates and quality of life [11].

### Contributing factors to breast cancer burden in India

Although the prevalence of breast cancer is steadily rising in India, many underlying issues contribute to this increasing burden [12]. Studies have shown that a low level of awareness and knowledge of breast cancer contributed to the delayed presentation of breast cancer among Indian women [13]. Due to India’s diverse structure, a significant disparity remains in awareness, education, access to healthcare, treatment affordability, and people’s overall perspective towards breast cancer [14]. Approximately 60% of breast cancer cases are diagnosed at stage III or IV as most women present to the hospital only when there is a noticeable secondary change like a large mass or changes to skin or chest wall, with minor symptoms being ignored [12]. Furthermore, the priority for healthcare is low and even in large cities, screening for breast cancer is alien to most people [15] and uptake of screening is low [16]. Therefore, the lack of screening programs and delay in diagnosis restrict improvements in breast cancer survival in India [17].

A lack of knowledge about breast cancer among Indian women is shown to be one of the main obstacles to early identification and screening [18]. A cross-sectional study including 260 Indian women, showed that 81% of women did not have knowledge of breast cancer and thought that Clinical Breast Examination by a doctor was the only option for breast cancer screening [19]. Another cross-sectional study showed that 85.1% of Indian women have never heard of breast cancer, 81.2% could not identify any symptoms and 87.7% were not able to state a single risk factor [20]. Awareness of early screening methods is also low in Indian women, with only 27.37% practising breast self-examination and only 7.12% taking up mammogram screening [18].

This qualitative web-based aimed to assess breast cancer survivor stories of Indian women to identify: (i) why Indian women were diagnosed at an advanced stage of breast cancer, (ii) the barriers that contributed to their delayed diagnosis, and (iii) the level of awareness and perceptions towards breast cancer. The objectives of this study were to: (i) identify secondary web-based data, in the form of blogs and YouTube videos, that provide insight into breast cancer survivor stories of Indian women, and (ii) analyse this identified data using inductive Thematic Analysis.

## Methods

This qualitative study used secondary web-based data to understand delayed diagnosis of breast cancer in Indian. Secondary web-based data provides access to a wide range of diverse voices [21], which is particularly important when studying a vast population like India. Resources such as blogs and YouTube videos provide a platform for breast cancer survivors to share their personal experiences, offering rich narratives that contribute to a deeper understanding of the patients’ experience [22]. Unlike interview formats which rely on establishing a dynamic between interviewer and interviewee: social media and blogs allow access to users’ personal accounts as publicly accessible artifacts [23]. Ethical Approval was granted by Brunel Research Ethics Committee (BREO) in July 2024 for the reuse of this openly available web-based secondary data (Reference: 49186-NER-Jul/2024- 51938-1).

### Screening of blogs and YouTube videos

In July 2024, the terms “Breast cancer survivor blogs by Indian women” were searched on Google to identify blogs; and “Breast cancer survivor stories by Indian women” and “Interviews of breast cancer survivors among Indian women” were searched on the YouTube search bar. 10 blogs and 21 YouTube videos were screened to see against inclusion criteria. Inclusion criteria for Blogs and YouTube videos included being authored by Indian breast cancer survivors, published in the last 10 years, reporting a delayed presentation of breast cancer symptoms, discussing reasons for delay in seeking medical attention. Blogs and videos in English and local languages including Hindi and Telugu were included. Hindi and Telugu were translated into English by the second author who is fluent in all of these languages. Exclusion criteria included blogs that required login access to read, not being based in India and not discussing reasons for delayed breast cancer presentation.

### Data collection and analysis

Blog texts were extracted for analysis. Manual transcription of spoken content in identified YouTube was performed. Participants’ anonymity was ensured throughout this study using pseudonyms, with any identifiers removed from data analysis. Demographic data of age, socioeconomic status and work status were extracted where reported by women within their accounts.

Identified blogs and YouTube video transcripts were analysed using inductive Thematic Analysis [24, 25]: with themes arising directly from the data without a pre-defined category or structure. The researcher familiarised themself with the data by reading and re-reading the transcribed texts from blogs and YouTube videos and actively listening to videos multiple times to gain an in-depth understanding. Notes from the initial reading were recorded in a Word document, including initial thoughts, patterns, and potential codes. Codes were identified and matched to all the data extracts using colour coding to ensure similar phrases and concepts were coded consistently. After coding and collating all data extracts, a mind map was generated of the main themes and sub-themes corresponding to more specific aspects of identified themes. The researcher then grouped the codes based on a common meaning, conceptual coherence, and a fundamental organising notion to create the themes. Themes were defined and refined, and an in-depth analysis of each theme was done to understand interesting aspects and an explanation of their significance [25].

## Results

Seventeen accounts from 5 blogs and twelve YouTube videos were included. All five blogs were written in English. Six YouTube videos were in Hindi, two in a mix of Hindi and English, one in English and three in Telugu. Ages of women ranged from 25 years to 49 years (ages 25–30: *n* = 3; 31–40: *n* = 6; 41–50: *n* = 3), with ages unspecified in five accounts. Of the seventeen accounts, two indicated that they were from a middle-class background and five indicated that they were working. Accounts discussed identification of breast cancer at stage I (*n* = 1; 5.9%), stage II (*n* = 1; 5.9%), stage III (*n* = 3; 17.6%) and stage IV (*n* = 4; 23.5%), ‘advanced stage’ (*n* = 2; 11.8%) and unspecified stage (*n* = 6; 35.3%). Symptoms reported as first identified by the women were lumps (*n* = 15; 88.2%), knots (*n* = 2; 11.8%), discharge (*n* = 2; 11.8%), pain and low energy (*n* = 1; 5.9%), with three women reporting multiple first symptoms. Supplementary File 1 provides sources of all identified accounts and data extraction.

Four main themes featuring a total of seven sub-themes were identified: (1) delay in recognising or responding to symptoms, with sub-themes of lack of knowledge, misinterpretation of symptoms and procrastination, (2) initial medical misjudgement, with sub-themes of dismissal of symptoms by doctors and false reassurance from medical tests, (3) socio-cultural factors, with sub-themes of family responsibilities and career responsibilities, (4) emotional and psychological factors (Table 1).

**Table 1:** Themes and sub-themes identified in identified blogs and YouTube videos

Theme	Summary
Delay in Recognising or responding to the symptoms	
Lack of knowledge	Insufficient awareness or understanding of breast cancer symptoms, leading to delayed presentation
Misinterpretation of symptoms	Symptoms are incorrectly interpreted
Procrastination	Deliberate postponement in addressing symptoms
Initial medical misjudgement	
Dismissal of symptoms by doctors	Healthcare professionals may downplay or overlook symptoms, contributing to a missed early detection of breast cancer
False reassurance from medical tests	Patients get a false sense of security from inaccurate or incomplete initial test results
Socio-cultural factors	
Family responsibilities	Women put the needs of their families before their own, leading to overlooking their health
Career responsibilities	Career commitments can cause delays in seeking or adhering to medical treatment
Emotional and psychological factors	Fear, anxiety or denial can hinder seeking timely medical attention

1. Delay in recognising or responding to the symptoms.

Thirteen out of seventeen women reported a delay in recognising or responding to their symptoms. Sub-themes identified that contributed to the delays included lack of knowledge, misinterpretation of symptoms, and procrastination.

A lack of knowledge regarding the early signs and symptoms, risk factors and seriousness of the symptoms of breast cancer, was prevalent in the included accounts. Many women initially underestimated the significance of a lump in their breasts: *“I felt a lump in my breast. I assumed I was imagining it and was worrying for no reason” (Blog 4)*. Two women reported that they did not know the cause of their symptoms: *“Unaware of its (the lump) cause*,* XX asked her relative for help” (Blog 1).* Participants often believed that they were not likely to develop breast cancer because of their age, healthy lifestyle, or lack of family history: *“I exercised five days a week and ate healthy. I had no family history of breast cancer” (Blog 5).* Additionally, some thought that lumps in the breast were normal in women: *“There’s a misconception that a lump is normal*,* so I thought it was normal. I never thought it could be cancer” (YouTube Video 3).*

Misinterpretation of early symptoms was identified as another key factor in diagnosis delay. Four women mistook early warning signs, such as lumps, for less serious conditions. A participant reported she assumed her lump was benign: *“I thought it was a benign tumour and took alternative therapy for it for three months thinking it would go away” (Blog 1)*, while another thought her lump could be due to hormonal changes: *“I convinced myself that this (the lump) was due to hormonal changes or because I’m just getting old” (YouTube Video 2).* Others misinterpreted their symptoms, attributing their lumps to clogged milk ducts during breastfeeding: *“I thought it was a clogged milk duct*,* so I ignored it” (YouTube Video 11).*

Procrastination was another key factor in diagnosis delay. Four women described ignoring or dismissing the seriousness of their symptoms: *“I felt a lump in my breast. I assumed I was imagining it and was worrying for no reason. I ignored it for a few days and continued with my daily commitments.” (Blog 4)*,* “I saw it (the lump)*,* but I did sit on it for some time*,* which I shouldn’t have” (YouTube Video 12).* They assumed their problem was minor or would go away on its own despite noticing a lump or other alarming signs: *“Initially*,* I thought it was something minor*,* but I completely ignored it. Even though I knew it could be a problem” (YouTube Video 2).*

2. Initial medical misjudgement.

Six out of seventeen women reported experiences of initial medical misjudgement. Six women reported that they received false reassurance from medical tests: *“It (ultrasound) didn’t reveal anything alarming” (Blog 2) or* were treated with a lack of urgency from their first medical visit: *“The doctor told me it was a normal lump and nothing to worry about” (YouTube Video 5).*

Dismissal of symptoms by healthcare professionals was a prominent sub-theme in the analysed accounts. Healthcare professionals often reassured them that their symptoms were benign: *“The doctor said it might be a benign lump and nothing else” (YouTube Video 3).* In addition, they did not think that breast cancer would occur at a young age: *“In general*,* at the age of 26*,* people (healthcare professionals) tend to think that nothing could be wrong*,* especially breast cancer of all things” (YouTube Video 1).* Doctors’ reassurances occasionally led patients to delay necessary tests, worsening their conditions: *“I emphasised and asked if it was cancer*,* and they said no*,* you can get a test done but there’s no hurry” (YouTube Video 5).*

False reassurance from medical tests was another identified sub-theme. Three women shared instances where first screenings, such as ultrasounds and mammograms, failed to detect abnormalities or provided inconclusive results: *“The mammogram results were clear*,* so the doctor said there’s nothing wrong and you are fine” (YouTube Video 7).* These false negatives contributed to further delay in diagnosis by making doctors and patients believe further investigation was unnecessary.

3. Socio-cultural factors.

Four out of seventeen women indicated that socio-cultural factors comprising family and career responsibilities played a crucial role in delaying their attention to breast cancer symptoms.

Family responsibilities leading to delayed diagnosis included women’s reported primary focus being fulfilling their roles as caregivers: *“I had itching and discharge but because my daughter was in 12th standard and was busy preparing for exams*,* I kept ignoring it. I was busy with my responsibilities” (YouTube Video 8).* Medical consultations were often postponed among these women due to the cultural expectations that family needs come before personal health: *“Let my son finish his 12th exam*,* then I will think on it (the lump). Typical Mother Responsibilities” (YouTube Video 12).* They felt that taking time off to seek medical advice would disrupt their caregiving duties.

Career responsibilities were described as leading to delayed diagnosis as women chose to ignore early symptoms alongside education and career commitments: *“I was preparing for some exams at the time I felt a lump*,* so my focus was completely on my preparation. I couldn’t give my attention to the symptoms” (YouTube Video 5)* and *“I was really busy with my PhD*,* and I was newly married” (YouTube Video 7)*.

4. Emotional and psychological factors.

Four out of seventeen women expressed emotional and psychological factors as contributing to delayed diagnosis: *“I kept denying that it can’t be cancer” (YouTube Video 5).* Despite recognising symptoms, women reporting delaying seeking medical attention due to fear and denial: *“I neglected it because I was scared that it might be cancer” (YouTube Video 2).* Additionally, they acknowledged the possibility of something being wrong when they noticed their symptom but preferred to avoid confronting it: *“I thought why will I get cancer” (YouTube Video 11).*

## Discussion

This study aimed to use secondary web-based data to identify reasons for delayed diagnosis of breast cancer, barriers contributing to delayed diagnosis and levels of awareness and perceptions of breast cancer in Indian women. Seventeen sources of data were identified and analysed using inductive Thematic Analysis. Four themes were identified using inductive Thematic Analysis from the blogs and YouTube videos: Delay in recognising or responding to symptoms, Initial medical misjudgement, Socio-cultural factors and Emotional and psychological factors.

The most prominent theme was the delay in recognising or responding to breast cancer symptoms, featuring three sub-themes: Lack of Knowledge, Misinterpretation of Symptoms, and Procrastination. Many women underestimated the significance of a lump or other symptoms due to a lack of knowledge about breast cancer risks. This misconception that they were not at risk led to a dangerous delay in seeking medical advice resulting in an advanced stage of breast cancer. Additionally, they were unaware of the cause and seriousness of the condition. A lack of knowledge about early breast cancer detection has been identified as a key influencer for increased cancer mortality in developing countries [26]. Misinterpretation or downplaying of symptoms, such as identifying a lump as a clogged milk duct when breastfeeding, have also been identified as key issues within primary research in developing countries [27] such as India [18] and Ghana [28]. A lack of government promotion to increase awareness of breast cancer symptoms is evident in India, whereby women are increasingly turning to social media for guidance on their symptoms [29]. These results underscore the vital need for improved public education to increase accurate awareness of breast cancer symptoms in India, emphasising the need for early detection and the fact that risk factors are not always apparent.

Another emergent theme from the data was initial medical misjudgement. Five participants’ symptoms were dismissed by healthcare providers often attributing them to benign conditions or underestimating the risk due to the patient’s age, which further delayed diagnosis and treatment. This is consistent with broader findings that doctors are more likely to dismiss breast cancer symptoms in younger women, as they believe that breast cancer is less common for them [30]. Indian healthcare professionals lack of awareness on key risk factors for breast cancer, such as age of menarche, age at menopause and late first pregnancy has also been identified in previous literature [31]. A literacy gap among health professionals in developing countries is a possible barrier to breast cancer prevention and early detection [32]. Furthermore, in two participants, ultrasound and mammogram tests failed to detect malignancies, providing patients with a false sense of security. Research has shown that mammograms and ultrasounds, while useful, are not infallible. Mammography can miss up to 20% of breast cancers, particularly in women with dense breast tissue, where the sensitivity of these tests is significantly reduced [33]. Furthermore, many tumours may go undetected by ultrasounds, although it is often the most available screening modality within the Indian healthcare system [34]. Therefore, there is a need for thorough interpretation and balance in assessments across these screening modalities. By acknowledging the limitations of diagnostic tools and avoiding assumptions based on patient demographics, healthcare providers can lessen the possibility of delayed diagnosis and improve outcomes for women at risk of breast cancer in India.

Women also reported sociocultural factors like the role of family and career responsibilities taking precedence over their health, ultimately leading to delays in addressing their symptoms. They described being focused on their children’s academic milestones or occupied with their careers and newly married life, rather than prioritising their health concerns. This finding highlights that societal pressures and gender roles can impede Indian women’s health-seeking behaviours. It emphasises the importance of establishing support systems that allow women to prioritise their health without feeling guilty or neglecting their familial or professional responsibilities. The societal and patriarchal obligations placed on Indian women to care for their husbands and children have a clear impact on their delayed presentation of breast cancer [35]. Women are hugely disadvantaged in the Indian healthcare system, with reduced access to diagnosis, resources and decision-making [36], accompanied by a stark gender divide between the government’s promotion of low-paid female community healthcare workers versus higher status male clinicians [37].

Emotional and Psychological Factors including fear and denial were found to play a significant role in delaying the diagnosis of breast cancer among breast cancer survivors in India. Four women admitted to avoiding seeking medical care or dismissing symptoms due to the fear of breast cancer diagnosis. Denial as a defence mechanism that helps individuals cope with the anxiety and fear associated with the possibility of having a serious illness like cancer [38]. Additionally, existing literature supports the idea that denial is a significant factor in delayed breast cancer diagnosis [39].

The findings from this study also reveal a striking gap in the uptake of routine breast cancer screening among the participants. Notably, only one out of the seventeen women mentioned performing breast self-examinations and none of the women discussed participating in regular breast cancer screening like mammograms. This is concerning, as early detection plays a critical factor in improving breast cancer outcomes. It highlights a significant area that requires more in-depth exploration through future research [17]. Furthermore, research should develop tailored educational interventions to raise awareness of the various types of breast cancer screening available, including self-examination, as well as encouraging the adoption of regular screening practices for breast cancer in India. Such educational interventions can be co-produced and delivered within the community by female Accredited Social Health Activists (ASHAs) for example [37]. Co-production between the public and health professionals can support the development of more culturally sensitive educational programs that can effectively bridge the knowledge gap and encourage proactive health-seeking behaviours among Indian women regarding breast cancer.

### Strengths and limitations

A strength of this study is its synthesises of secondary data providing personal accounts of breast cancer diagnosis in Indian women survivors of breast cancer. Despite the valuable insights gained from this study, there are certain limitations. This study relies on self-reported data from blogs and YouTube, which can be subject to recall bias or inaccuracies in participants’ recollections of their experiences and diagnosis timelines [40]. Reuse of secondary web-based information means that clarification on context or unclear information cannot be sought from the individual in question, as opposed to primary data collection such as interviews [41]. Additionally, individuals who choose to share their experiences online may not be representative of an overall population, limiting generalisability [41]. Identified sources were written or spoken in Hindi, English and Telugu: languages spoken in areas of India with identified higher literacy and socioeconomic status [42]. Additionally, there could be a potential loss of nuanced meaning during the translation of Hindi and Telugu YouTube videos used in this study. To capture a broader range of experiences and outcomes, future research should consider conducting primary qualitative interviewing as it may offer a more comfortable setting for breast cancer survivors to share their unfiltered experiences, thereby increasing data variability. Nevertheless, the results align with previous research, supporting the idea that the results can be generalised to an extent to the diverse Indian population.

## Conclusion

This study used existing blogs and YouTube videos to identify the multifaceted barriers leading to delayed breast cancer diagnosis in Indian women. Identified factors included delays in recognising or responding to the symptoms, initial medical misjudgement, sociocultural factors, and emotional and psychological challenges, revealing the complex interplay of personal, societal, and systemic issues in delaying breast cancer diagnosis among Indian women. These findings highlight the urgent need for targeted interventions to increase awareness of breast cancer symptoms and screening methods, improve healthcare provider training, and develop culturally sensitive support systems for Indian women. By addressing these barriers, it may be possible to improve the early detection and treatment outcomes for breast cancer in India, thereby reducing cancer mortality rates and improving the quality of life of Indian women diagnosed with breast cancer.

## Supplementary Information

Below is the link to the electronic supplementary material.

Supplementary Material 1

## Data Availability

Details on the secondary web-based data used is provided within the manuscript and supplementary file.
